# Diagnostic and prognostic value of neutrophil gelatinase-associated lipocalin, matrix metalloproteinase-9, and tissue inhibitor of matrix metalloproteinases-1 for sepsis in the Emergency Department: an observational study

**DOI:** 10.1186/s13054-014-0634-6

**Published:** 2014-11-19

**Authors:** Miaomiao Wang, Qian Zhang, Xin Zhao, Guijuan Dong, Chunsheng Li

**Affiliations:** Emergency Department, Beijing Chao-yang Hospital, Capital Medical University, 8# Worker’s Stadium South Road, Beijing, Chao-yang District 100020 China

## Abstract

**Introduction:**

The aim of this study was to evaluate the early diagnostic, risk stratification and prognostic value of neutrophil gelatinase-associated lipocalin (NGAL), matrix metalloproteinase-9 (MMP-9) and tissue inhibitor of matrix metalloproteinases-1 (TIMP-1), compared with procalcitonin (PCT) and the Mortality in Emergency Department Sepsis (MEDS) score in septic patients in the emergency department (ED).

**Methods:**

In total, 480 consecutive adult patients were enrolled in this study. They fulfilled the systemic inflammatory response syndrome (SIRS) criteria and were admitted to the ED of Beijing Chaoyang Hospital from February 2013 to August 2013. A total of 40 healthy controls comprised the control group. The patients were classified into four groups: SIRS, sepsis, severe sepsis, and septic shock. Serum NGAL, MMP-9, TIMP-1 and PCT were measured, and MEDS score was calculated at enrollment. The prognostic values of NGAL, MMP-9 and TIMP-1 were compared with PCT and MEDS score. A 28-day follow-up was performed for all patients.

**Results:**

The median levels of serum NGAL and TIMP-1 increased with sepsis severity. The areas under the receiver operating characteristic (AUC) curves of NGAL or TIMP-1 were greater than those of PCT and MEDS score in diagnosing and predicting 28-day mortality, and the AUC of a combination of NGAL and MEDS score or TIMP-1 and MEDS score was more significant. Serum NGAL, MMP-9 and TIMP-1 levels were significantly higher in non-survivors than survivors at 28 days’ follow-up. In addition, the level of NGAL was much higher in septic patients with acute kidney injury (AKI) than those without AKI. NGAL, TIMP-1, MMP-9 and MEDS score were found to be independent predictors of 28-day mortality in septic patients. The levels of serum NGAL and TIMP-1 were positively correlated with PCT and MEDS score in every septic group.

**Conclusions:**

NGAL and TIMP-1 are valuable for the risk stratification, early diagnosis and prognostication of sepsis in the ED. NGAL is also a valuable biomarker for prognosis of septic patients with AKI in the ED.

## Introduction

Sepsis is the systemic inflammatory response to infection, and has a high mortality rate despite the use of modern antibiotics and resuscitation therapies [1], particularly in septic patients with associated acute kidney injury (AKI). Early diagnosis, appropriate classification and intervention in the initial periods of sepsis play a crucial role in decreasing mortality, and they are implemented in the emergency department (ED) [2]. Although clinical scoring systems are useful tools to evaluate the severity and prognosis of sepsis, most of them are created for ICU patients and may not be applicable to the ED. Finding biomarkers and combining them with the clinical scoring systems for early diagnosis, risk stratification, and evaluation of prognosis of sepsis has great significance.

Neutrophil gelatinase-associated lipocalin (NGAL) is an endogenous bacteriostatic protein that is expressed and secreted by neutrophils, macrophages, hepatocytes, and renal tubular cells in various pathologic states [3,4]. NGAL represents the activities of the neutrophils and comprises a critical component of innate immunity to bacterial infection [5]. Matrix metalloproteinase-9 (MMP-9) and its inhibitor, tissue inhibitor of matrix metalloproteinase-1 (TIMP-1), are promising novel biomarkers to predict the severity and outcome of sepsis [6,7], and are involved in the pathogenesis of sepsis and septic shock [8]. NGAL seems to positively modulate the activity of MMP-9 [9]. It protects MMP-9 from proteolytic degradation and enhances its enzymatic activities by binding and forming the MMP-9/NGAL complex.

Some clinical studies have confirmed that serum NGAL, MMP-9 and TIMP-1 levels are increased in septic patients [10,11]. However, most of the previous studies, which had small sample sizes, included ICU admission patients, and the relationship of these biomarkers could not be expounded. In this study, we questioned whether serum NGAL, MMP-9 and TIMP-1 concentrations are different according to the severity of sepsis, and compared their clinical value in early diagnosis, risk stratification and prognostic evaluation of sepsis with procalcitonin (PCT) and the mortality in emergency department sepsis (MEDS) score.

## Materials and methods

### Patients and grouping

A single-center observational study was conducted in the ED of Beijing Chaoyang Hospital, an urban university tertiary hospital with approximately 250,000 ED admissions per year. Between February 2013 and August 2013, consecutive patients who fulfilled the systemic inflammatory response syndrome (SIRS) criteria defined by the American College of Chest Physicians/Society of Critical Care Medicine (ACCP/SCCM) were enrolled [12]. Forty healthy individuals comprised the age-matched control group from volunteers in the Physical Examination Center of Beijing Chao-yang Hospital. Patients were classified at the time of enrollment as having SIRS, sepsis, severe sepsis, or septic shock, according to ACCP/SCCM criteria [12]. Antibiotics and fluid resuscitation were the mainstays of therapy for patients with severe sepsis. Other interventions, including vasoactive agents and mechanical ventilation, were administered if necessary.

The exclusion criteria were as follows: patients aged <18 years, and patients (or their relatives) who declined to participate. Four hundred and forty patients were enrolled and followed up for 28 days or until death. This study was approved by the Beijing Chao-yang Hospital Ethics Committee. Written informed consent was obtained from all participants (or their relatives) [13].

### Measurement methods

Subject data, name, age, sex, past medical history and vital signs were recorded immediately at enrollment. Some correlative laboratory examinations were carried out and recorded within 24 hours. Venous blood samples were obtained at the time of ED admission, collected in tubes containing heparin or ethylenediamine tetraacetate, separated by centrifugation at 3,000 rpm for 5 minutes, and stored at –80°C until assayed. The levels of NGAL, MMP-9 and TIMP-1 were determined by enzyme-linked immunosorbent assay using commercially available kits (R&D Systems Inc., Minneapolis, MN, USA). The NGAL, MMP-9 and TIMP-1 reference ranges were 100 to 300 ng/mL, 20 to 200 ng/mL, and 150 to 500 ng/mL, respectively. PCT was measured using a bioMerieux Mini VIDAS® immunoassay analyzer (Block Scientific, Bohemia, NY, USA) using serum samples. And the upper and lower detection limits of PCT were 200 ng/mL and 0.05 ng/mL. The MEDS score was calculated when the patients were admitted to the ED according to age, past medical history, vital signs and laboratory results [14]. Septic patients were classified into surviving and non-surviving groups according to 28-day survival, and those who died from all causes within the follow-up time were considered non-survivors. In addition, the septic patients were observed to see whether they developed AKI, which was defined as an abrupt (within 48 h) decrease in kidney function, with an absolute increase in serum creatinine ≥0.3 mg/dL or a percentage increase in serum creatinine ≥50% (1.5-fold from baseline), according to the Acute Kidney Injury Network (AKIN) criteria [15].

### Statistical analysis

All data were analyzed using SPSS 16.0 software (SPSS Inc., Chicago, IL, USA). Non-normally distributed data, including serum NGAL, MMP-9 and TIMP-1 levels, serum PCT levels, and MEDS score, were expressed as the median (25th to 75th percentile). Kruskal-Wallis one-way analysis of variance was applied for multi-group comparisons, and two-group comparisons were performed using the Mann-Whitney *U*-test. To compare the prediction of NGAL, MMP-9, TIMP-1, PCT, and MEDS score for 28-day mortality, receiver operating characteristic (ROC) curves were constructed and the area under the ROC curve (AUC) was determined. On the basis of optimal thresholds determined according to ROC curve analysis, prognostic parameters, including sensitivity, specificity, positive predictive value (PPV) and negative predictive value (NPV), were also calculated. In addition, the *Z*-test formula was applied for comparisons of AUCs. Binary logistic regression analysis was applied to determine the independent predictors of 28-day mortality. All statistical tests were two-tailed, and *P* <0.05 was considered statistically significant. Finally, Spearman correlation analysis was applied to determine the correlation between NGAL, MMP-9, TIMP-1 and MEDS score.

## Results

### Patient demographics

There were no significant differences in age and sex among the five groups of enrolled subjects (SIRS, sepsis, severe sepsis, septic shock, and control groups) (Table 1). The baseline characteristics, diseases and associated infections in the enrolled subjects are also presented in Table 1. The 28-day mortality was 31.1% in SIRS and septic patients. The 28-day mortality increased with clinical severity of sepsis, and the differences between the two groups were significant (*P* <0.01).

**Table 1:** **Patient characteristics**

	**Control**	**SIRS**	**Sepsis**	**Severe sepsis**	**Septic shock**	***P***
Number	40	80	180	90	90	
Age, years	68 (65 to 74)	70 (58 to 76)	71 (59 to 78)	73 (60 to 78)	73 (65 to 78)	0.101
Male	57.0%	53.8%	61.2%	62.9%	58.1%	0.487
White blood cells, ×10^9^/L	6.8 (4.6 to 7.9)	9.5 (7.0 to 11.3)	13.5 (11.2 to 15.3)	14.1 (11.3 to 18.5)	16.6 (13.3 to 22.4)	0.002
Neu, ×10^9^/L	4.7 (3.0 to 6.2)	7.9 (5.3 to 9.4)	10.15 (8.8 to 12.1)	12.9 (9.2 to 15.6)	13.9 (11.4 to 18.9)	0.003
Procalcitonin, ng/mL	0.13 (0.06 to 0.31)	0.38 (0.48 to 1.19)	1.53 (1.23 to 4.45)	5.76 (2.45 to 9.43)	8.86 (0.88 to 38.96)	<0.001
MEDS score	0	4.28 (2.24 to 6.34)	6.7 (5.34 to 8.23)	18.17 (15.1 to 21.3)	24.28 (22.89 to 26.12)	<0.001
NGAL, g/mL	197.8 (178.5 to 208.8)	224.7 (200.3 to 243.5)	239.1 (223.6 to 256.8)	251.0 (233.4 to 262.2)	264.4 (242.8 to 282.4)	0.002
MMP-9, ng/mL	60.4 (51.9 to 73.4)	74.9 (62.1 to 82.4)	83.7 (66.5 to 97.7)	96.9 (76.5 to 116.8)	83.6 (62.0 to 96.6)	0.093
TIMP-1, ng/mL	228.2 (205.4 to 242.3)	247.4 (230.5 to 265.4)	262.6 (240.6 to 282.7)	279.3 (243.4 to 296.1)	321.8 (287.4 to 346.4)	0.001
MMP-9/TIMP-1	0.27 (0.20 to 0.33)	0.30 (0.24 to 0.35)	0.32 (0.26 to 0.38)	0.35 (0.25 to 0.45)	0.26 (0.17 to 0.35)	0.128
Blood urea nitrogen, mmol/L	5.68 (3.88 to 7.89)	6.04 (3.92 to 8.04)	9.48 (7.80 to 11.56)	11.27 (9.92 to 14.04)	12.56 (10.84 to 15.12)	0.079
Serum creatinine, mg/dL	0.68 (0.43 to 0.82)	0.73 (0.52 to 0.97)	1.56 (.0.83 to 1.89)	2.45 (0.98 to 5.20)	2.63 (0.87 to 6.16)	0.063
28-day mortality, n (%)		11 (13.8%)	31 (17.3%)	38 (42.2%)	57 (63.0%)	<0.001
Mortality in ED, n (%)		0	1 (0.6%)	6 (6.7%)	14 (15.6%)	<0.001
Mechanical ventilation		0	0	35 (38.9%)	68 (75.6%)	<0.001
in ED, n (%)						
**Main diagnosis** n (%)						
Pneumonia		62 (77.5%)	132 (73.3%)	61 (67.8%)	70 (77.8%)	0.481
IAI		8 (10.0%)	28 (15.6%)	16 (17.8%)	14 (15.6%)	0.665
USI		5 (6.25%)	7 (3.89%)	7 (7.78%)	2 (2.22%)	0.723
CNSI		3 (3.75%)	8 (4.44%)	5 (5.56%)	4 (4.44%)	0.558
SSTI		0	4 (2.22%)	1 (1.11%)	0	
DKA		2 (2.50%)	1 (0.56%)	0	0	

The values are expressed as median (IQR, observations available) or number (percentage). MEDS, mortality in emergency department sepsis; NGAL, neutrophil gelatinase-associated lipocalin; SIRS, systemic inflammatory response syndrome; MMP-9, matrix metalloproteinase-9; TIMP-1, tissue inhibitor of matrix metalloproteinase-1; ED, emergency department; IAI, intra-abdominal infection; USI, urinary system infection; CNSI, central nervous system infection; SSTI, skin/soft tissue infection; DKA, diabetic ketoacidosis.

### Comparison of median levels of NGAL, MMP-9, TIMP-1 and MMP-9/TIMP-1 ratio, and MEDS score

**Figure 1:**
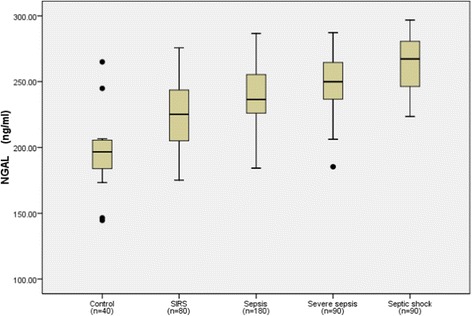
**Neutrophil gelatinase-associated lipocalin (NGAL) levels in healthy control individuals, and patients with systemic inflammatory response syndrome (SIRS), sepsis, severe sepsis and septic shock on emergency department admission.** Lines denote median values, boxes represent 25th to 75th percentiles and whiskers indicate the range. Numbers of samples are indicated in parentheses.

**Figure 2:**
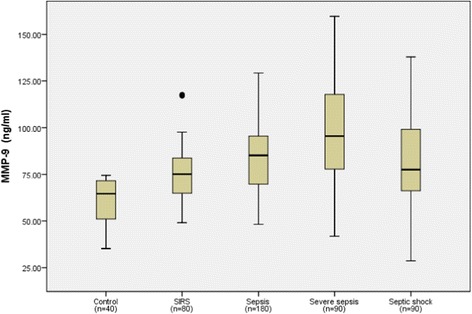
**Matrix metalloproteinase-9 (MMP-9) levels in healthy control individuals, and patients with systemic inflammatory response syndrome (SIRS), sepsis, severe sepsis and septic shock on emergency department admission.** Lines denote median values, boxes represent 25th to 75th percentiles and whiskers indicate the range. Numbers of samples are indicated in parentheses.

**Figure 3:**
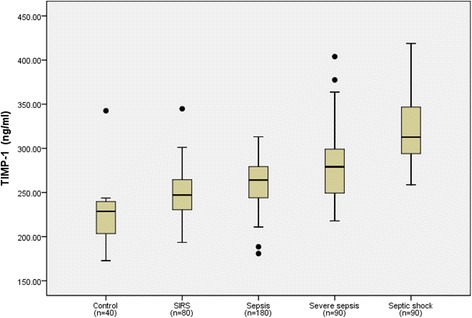
**Tissue inhibitor of matrix metalloproteinase-1 (TIMP-1) levels in healthy control individuals, and patients with systemic inflammatory response syndrome (SIRS), sepsis, severe sepsis and septic shock on emergency department admission.** Lines denote median values, boxes represent 25th to 75th percentiles and whiskers indicate the range. Numbers of samples are indicated in parentheses.

The median NGAL, MMP-9, TIMP-1 and MMP-9/TIMP-1 ratio levels, the PCT levels, and MEDS scores in each group are shown in Table 1 and NGAL, MMP-9, TIMP-1 levels, in each group are also shown in Figures 1, 2 and 3. Serum NGAL, TIMP-1, PCT and MEDS score at ED admission were significantly different among the groups. Compared with the healthy control group, NGAL, MMP-9 and TIMP-1 levels were significantly higher in patients with SIRS, sepsis, severe sepsis and septic shock (*P* <0.01). In addition, NGAL, MMP-9, TIMP-1 levels and MEDS score were significantly higher in sepsis, severe sepsis and septic shock than in SIRS (*P* <0.01). NGAL, TIMP-1, PCT levels and MEDS score were markedly higher in severe sepsis and septic shock than in sepsis (*P* <0.01), and were clearly higher in septic shock than in severe sepsis (*P* <0.01). However, the MMP-9 and MMP-9/TIMP-1 ratio was not significantly higher in severe sepsis and septic shock than in sepsis, or in septic shock than in severe sepsis.

### NGAL, MMP-9 and TIMP-1 levels in survivors and non-survivors

**Figure 4:**
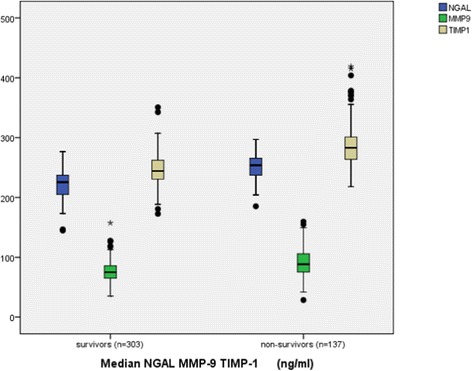
**Neutrophil gelatinase-associated lipocalin (NGAL), matrix metalloproteinase-9 (MMP-9) and** t**issue inhibitor of matrix metalloproteinase-1 (TIMP-1) levels in survivors and non-survivors for 28-day mortality in systemic inflammatory response syndrome (SIRS) and septic patients.** Lines denote median values, boxes represent 25th to 75th percentiles and whiskers indicate the range. Numbers of samples are indicated in parentheses. The median NGAL (blue), MMP-9 (green) and TIMP-1 (brown) levels were higher in non-survivors than in survivors (all *P* <0.001).

NGAL, MMP-9 and TIMP-1 levels were higher in non-survivors than in survivors (all *P* <0.01). As shown in Figure 4, PCT was also higher in non-survivors than survivors (*P* <0.01).

### Value of NGAL, MMP-9, TIMP-1 and MEDS score for predicting 28-day mortality

The AUC of NGAL for predicting 28-day mortality in septic patients was 0.833, and of TIMP-1 was 0.845, higher than that of PCT (0.768; *P* <0.05) or MEDS score (0.763; *P* <0.05). The AUC of MMP-9 for predicting 28-day mortality in septic patients was 0.700, lower than that for the PCT and MEDS score.

**Figure 5:**
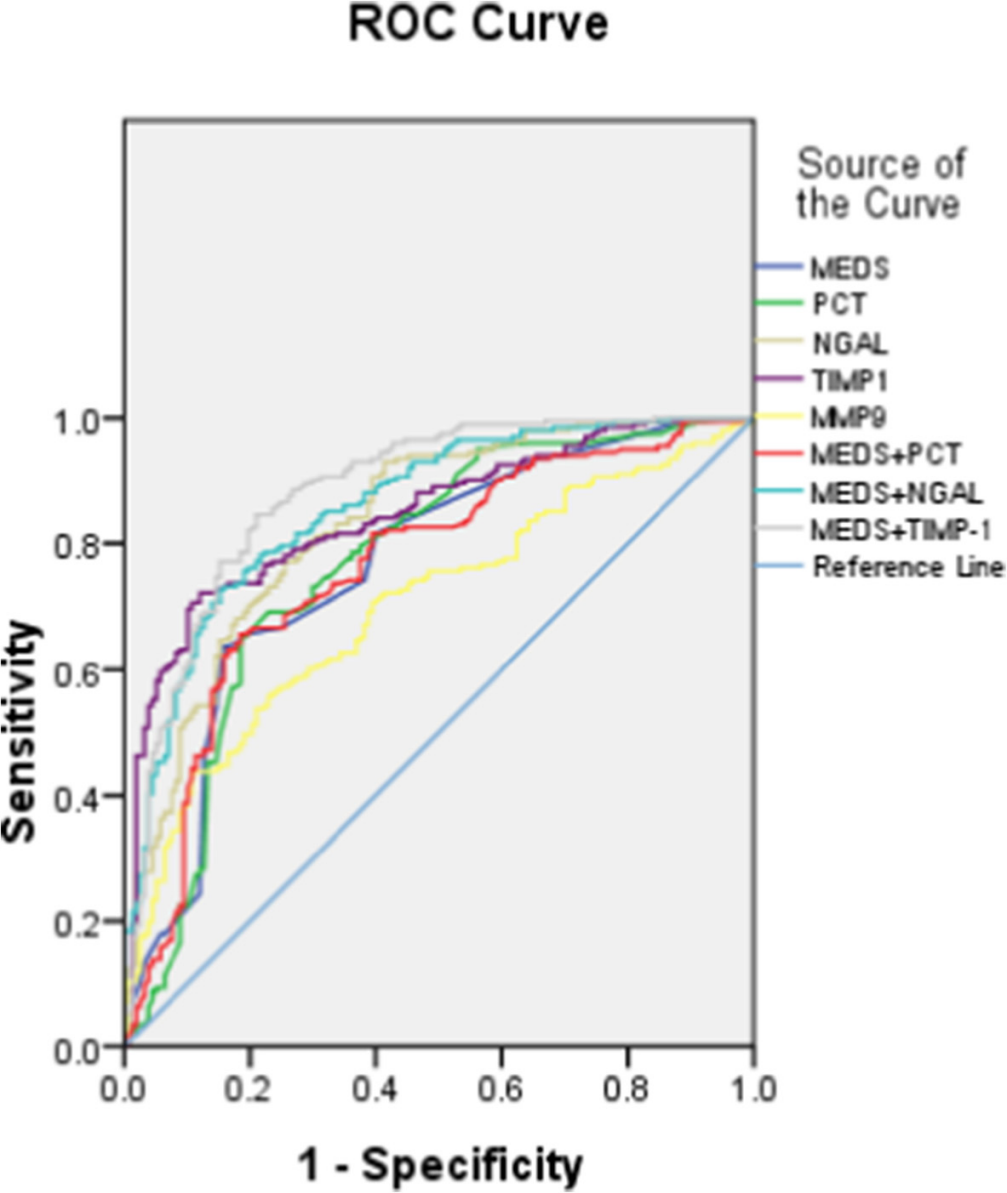
**Receiver operating characteristic curves for mortality in emergency department sepsis (MEDS) score, procalcitonin (PCT), neutrophil gelatinase-associated lipocalin (NGAL), tissue inhibitor of matrix metalloproteinase-1 (TIMP-1), matrix metalloproteinase-9 (MMP-9), MEDS + PCT, MEDS + NGAL and MEDS + TIMP-1 for predicting 28-day mortality in septic patients.**

The AUC of NGAL and TIMP-1 in combination with the MEDS score was 0.858 and 0.882, respectively, which was statistically significant compared with PCT alone (0.768; all *P* <0.05) or the combination of PCT and MEDS score (0.782; all *P* <0.05). The ROC curves of these biomarkers for predicting 28-day mortality among the five groups are shown in Figure 5.

Using a NGAL cutoff value of 236.62 ng/mL for predicting 28-day mortality in septic patients, the sensitivity was 71.8%, specificity was 77.1%, the PPV was 79.5%, and NPV was 74.5%. Using a TIMP-1 cutoff value of 268.27 ng/mL for predicting 28-day mortality in septic patients, the sensitivity was 71.1%, specificity was 88.4%, the PPV was 72.1%, and the NPV was 87.9%. The detailed results are presented in Tables 2 and 3.

**Table 2:** **Area under the curve of various parameters for predicting 28-day mortality in septic patients and for septic acute kidney injury (AKI)**

	**Variable**	**AUC**	**Standard error**	***P*** **-value**	**95% Confidence interval**	
					**Lower limit**	**Upper limit**
28-day mortality	MEDS	0.763	0.026	<0.001	0.713	0.814
	PCT	0.768	0.026	<0.001	0.716	0.819
	NGAL	0.833	0.021	<0.001	0.791	0.875
	MMP-9	0.700	0.027	<0.001	0.646	0.754
	TIMP-1	0.845	0.020	<0.001	0.805	0.885
	NGAL + MEDS^a^	0.858	0.020	<0.001	0.820	0.896
	TIMP-1 + MEDS^a^	0.882	0.018	<0.001	0.846	0.918
	PCT + MEDS^a^	0.782	0.026	<0.001	0.721	0.817
Septic AKI	NGAL	0.897	0.022	<0.001	0.853	0.940
	TIMP-1	0.647	0.041	<0.001	0.568	0.727
	PCT	0.645	0.038	<0.001	0.571	0.719

^a^Compared with mortality in emergency department sepsis (MEDS) score, P < 0.01. AUC, area under the receiver operating characteristic curve; PCT, procalcitonin; NGAL, neutrophil gelatinase-associated lipocalin; MMP-9, matrix metalloproteinase-9; TIMP-1, tissue inhibitor of matrix metalloproteinase-1.

**Table 3:** **Performance of multivariable models for predicting 28-day mortality in septic patients**

	**Variable**	**Cutoff**	**Sensitivity, %**	**Specificity, %**	**PPV, %**	**NPV, %**
28-day mortality	NGAL, ng/mL	236.62	71.80	77.10	79.50	74.50
	MMP-9, ng/mL	95.29	55.30	83.80	43.80	89.20
	TIMP-1, ng/mL	268.27	74.10	88.40	72.10	87.90
	MMP-9/TIMP-1 ratio	0.401	47.70	77.80	20.90	92.40
	PCT, ng/mL	4.27	64.30	84.10	65.20	84.10

NGAL, neutrophil gelatinase-associated lipocalin; MMP-9, matrix metalloproteinase-9; TIMP-1, tissue inhibitor of matrix metalloproteinase-1; PCT, procalcitonin;

NPV, negative predictive value; PPV, positive predictive value.

### NGAL, MMP-9, TIMP-1, MEDS score as independent predictors of 28-day mortality

Age, sex, NGAL, MMP-9, TIMP-1, PCT and the MEDS score were included in a multivariate logistic regression model to determine the independent predictors of 28-day mortality. Using binary logistic regression analysis, NGAL (B = 0.047, odds ratio (OR) = 1.048, *P* = 0.010), MEDS score (B = 0.124, OR = 1.132, *P* <0.001), TIMP-1 (B = 0.039, OR = 1.040, *P* <0.001) and MMP-9 (B = 0.020, OR = 1.020, *P* = 0.010) were found to be independent predictors of 28-day mortality in septic patients, but PCT (B = 0.109, OR = 0.894, *P* = 0.010), age and gender were not (Table 4).

**Table 4:** **Independent factors predicting 28-day mortality in septic patients**

	**Variable**	***B***	**Standard error**	**Wald**	***P*** **-value**	**Odds ratio**	**95% CI for EXP (** ***B*** **)**	
							**Lower limit**	**Upper limit**
28-day mortality	MEDS	0.124	0.030	17.595	<0.001	1.132	1.068	1.200
	PCT	-0.112	0.043	6.589	0.010	0.894	0.821	0.974
	NGAL	0.047	0.010	22.117	<0.001	1.048	1.028	1.068
	MMP-9	0.020	0.008	6.153	0.013	1.020	1.004	1.037
	TIMP-1	0.039	0.007	28.305	<0.001	1.040	1.025	1.055
	Constant	-24.018	2.964	65.657	<0.001	0.000		

MEDS, Mortality in Emergency Department Sepsis. PCT, procalcitonin; NGAL, neutrophil gelatinase-associated lipocalin; MMP-9, matrix metalloproteinase-9; TIMP-1, tissue inhibitor of matrix metalloproteinase-1.

### Correlation of serum NGAL levels with serum MMP-9, TIMP-1 and PCT levels

Spearman correlation analysis of NGAL with neutrophils, MMP-9, TIMP-1 and PCT showed correlation coefficients of 0.602, 0.302, 0.583, and 0.526, respectively (all *P* <0.001). The correlation coefficient between MMP-9 and TIMP-1 was 0.391, which suggested positive correlation.

### NGAL levels in AKI and non-AKI septic patients

**Figure 6:**
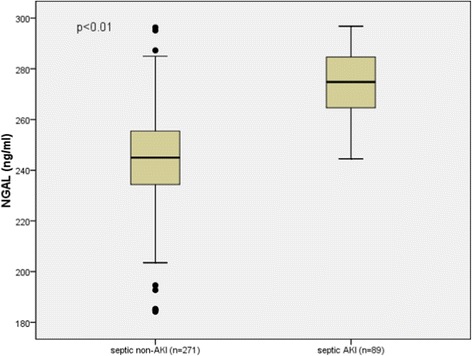
**Neutrophil gelatinase-associated lipocalin (NGAL) levels in non-acute kidney injury (AKI) and AKI of septic patients.** Lines denote median values, boxes represent 25th to 75th percentiles and whiskers indicate the range. Numbers of samples are indicated in parentheses.

**Figure 7:**
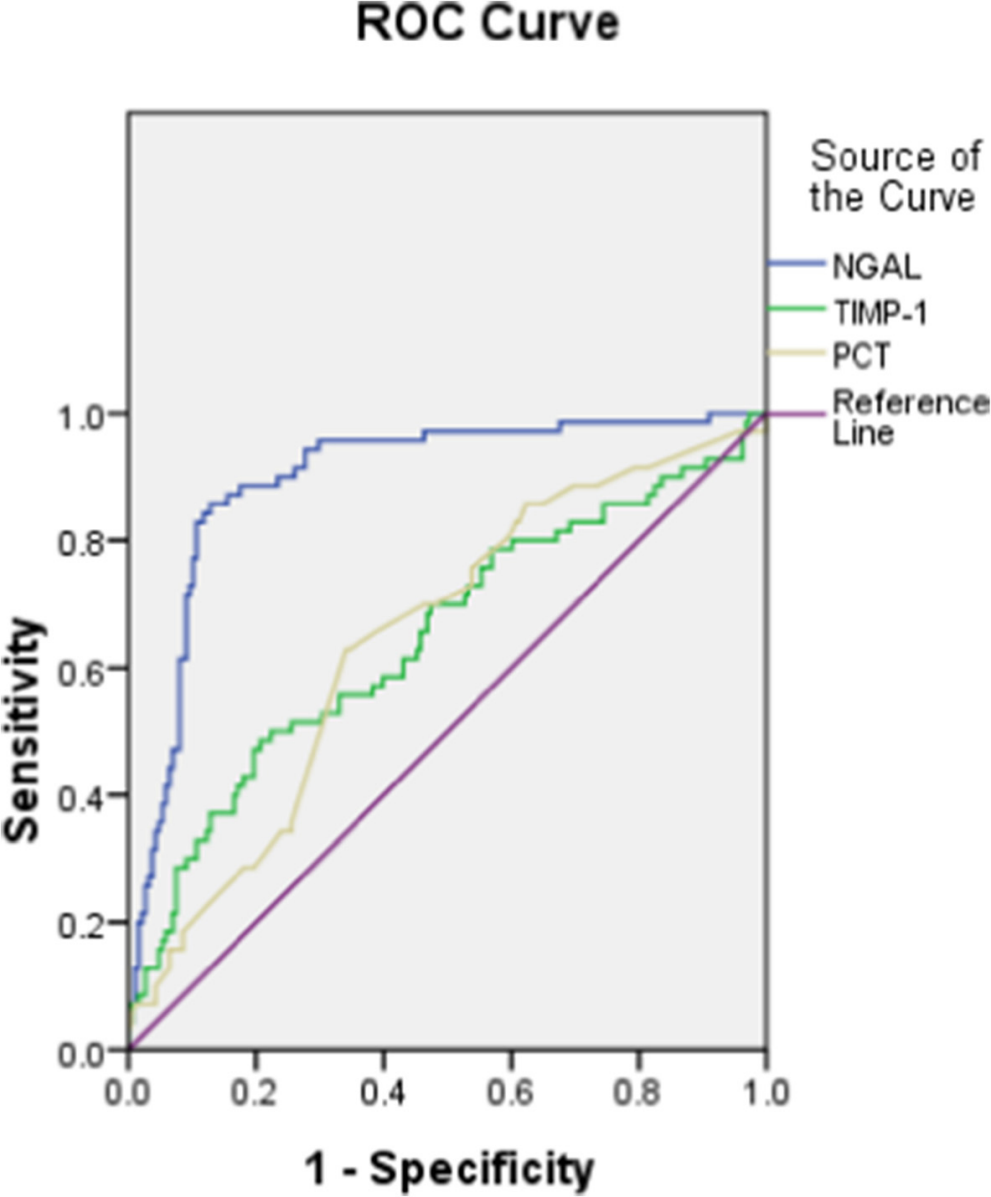
**Receiver operating characteristic curves of neutrophil gelatinase-associated lipocalin (NGAL), tissue inhibitor of matrix metalloproteinase-1 (TIMP-1) and procalcitonin (PCT) for diagnosis of acute kidney injury (AKI) in sepsis.**

The NGAL level was higher in AKI than in non-AKI septic patients (*P* <0.01). As shown in Figure 6, the AUC of NGAL for predicting AKI in septic patients was 0.897, higher than that of PCT (0.645; *P* <0.01) and TIMP-1 (0.647; *P* <0.01) (Figure 7 and Table 2).

## Discussion

During recent decades, the mortality of sepsis has remained unacceptably high. Early diagnosis and exact risk stratification, which are keys to initiating timely treatments, are significant predictors [1]. Consequently, there is a demand for useful severity scoring systems and biomarkers to improve the outcomes in early diagnosis and risk assessment. Although it has disadvantages compared with the other severity scoring systems, the MEDS score is derived from septic patients in the ED and is more suitable for predicting mortality in this cohort [16,17]. In the present study we chose MEDS score as a comparative variable to NGAL and TIMP-1. Meanwhile, in diagnosis, differential diagnosis, risk stratification and prognosis biomarkers play important roles in septic patients. The most widely used clinical biomarker is PCT, and while confirmed to be superior to other biomarkers in some studies, its prognostic value is controversial [18,19].

Consistent with previous studies [20,21], our study indicates the NGAL is elevated in the peripheral blood of sepsis patients. Human NGAL was originally identified as a novel protein isolated from secondary granules of human neutrophils and was subsequently demonstrated to be a 25-kDa protein covalently bound to neutrophil gelatinase [3,22]. NGAL protein is mainly synthesized at the early-myelocyte stage of granulopoiesis during formation of secondary granules, and NGAL mRNA is normally expressed in many adult human tissues, such as bone marrow, uterus, prostate, salivary gland, stomach, colon, trachea, lung, liver and kidney [23]. In addition, NGAL comprises a critical component of innate immunity to bacterial infection. Siderophores are synthesized by bacteria to scavenge iron from their surroundings, and use specific transporters to recover the siderophore-iron complex, ensuring their iron supply. The siderophore-chelating property of NGAL therefore renders it a bacteriostatic agent [24]. NGAL expression has been studied in several normal tissues where it functions to modulate oxidative stress and to provide protection against bacterial infection, and it has been proved in animal experiments [25]. Based on these two mechanisms, it is not surprising that circulating NGAL is increased in sepsis because of its structure and function, and that NGAL level increased according to the clinical severity of sepsis in our study.

MMP-9 is one of the family of MMPs, and is induced by many inflammatory factors, including interleukin (IL)-1b, IL-8, and tumor necrosis factor-α. It is stored in the tertiary granules of polymorphonuclear leukocytes, which are key effectors in acute inflammatory diseases such as sepsis [26]. MMPs are not expressed during normal conditions but are expressed and activated increasingly during inflammation, and their main function has been considered to be the degradation and removal of extracellular matrix molecules from the tissue [27]. It is reported that MMP-9 and gelatinase activity increased significantly after sepsis, and TIMP-1, an MMP-9 inhibitor, blocked these activities, as well as the ensuing septic shock. Furthermore, with the development of sepsis, these results are consistent with MMP-9-induced caspase-3 activation in response to infection, which increases TIMP-1 level and thereby inhibits MMP-9, in turn decreasing transforming growth factor-β1 and caspase-3 signaling pathways and improving survival in septic rats [28]. The integrity of tissue architecture is closely dependent on the delicate balance between the activation of MMPs and their inhibition by TIMPs. Any alteration in this balance is linked to tissue damage. Elevated levels of MMP-9 and TIMP-1 were reported in septic patients, and higher TIMP-1 levels at the beginning of severe sepsis were predictive of death [29].

In humans, NGAL was originally identified as a 25-kDa protein covalently linked to MMP-9 in human neutrophils, which normally provide the main cellular source of circulating NGAL. By forming the MMP-9/NGAL complex, NGAL protects MMP-9 from proteolytic degradation, increasing the enzymatic activity of MMP-9 and subsequently enhancing tumoral invasiveness and diffusion [30]. In the present study, MMP-9 was higher with increased severity of sepsis in the groups with SIRS, sepsis and severe sepsis (*P* <0.01), but the level decreased according to severity of septic shock. MMP-9 elevation has become a useful marker of the severity of sepsis [10], but a multicenter study by Lorente *et al*. [31] found a non-significant increase in MMP-9. MMP/TIMP ratios may mirror the balance between the proteolytic activity of MMPs and their tissue inhibitors, and the importance of reduced MMP-9/TIMP-1 ratio as a predictive biomarker of severity in septic patients [31]. In our study, TIMP-1 levels significantly increased on admission according to the severity of sepsis. Therefore, the variation in MMP-9 was not only related to the change in TIMP-1, which is the specific inhibitor of MMP-9, but also to the variation of NGAL.

The ED often serves as the first location for initial evaluation and risk stratification, which is essential to the timely management of septic patients. Our research also focuses on the early prognostic evaluation of septic patients. Our study suggests that the prognostic value of NGAL, MMP-9 or TIMP-1 is superior to PCT and MEDS score, and that NGAL, TIMP-1, MMP-9 or MEDS score, but not PCT, are robust independent predictors of 28-day death in patients with sepsis. We found that the difference in NGAL, TIMP-1 and MMP-9 levels between non-survivors and survivors was significant. From the ROC analysis of a large sample of septic patients at ED admission, we found that NGAL and TIMP-1 were superior to PCT and showed higher sensitivity, specificity, PPV, and NPV in the early diagnosis of 28-day mortality. Taken together, these results suggest that NGAL and TIMP-1 may be useful to identify septic patients at risk of short-term mortality in the ED.

The clinical practice scoring systems are useful tools to evaluate the severity and prognosis of septic patients. While severity scoring systems and biomarkers have their own advantages and disadvantages in the prognosis of sepsis, both can be considered objective and accurate. Researchers have attempted to combine severity scoring systems with biomarkers to improve the accuracy of risk stratification and prognosis. In our study, the prognostic ability of the combination of NGAL or TIMP-1 with MEDS score was superior to either biomarker or MEDS score alone, and also superior to the combination of PCT with MEDS score. The combination of severity scoring systems and biomarkers is more effective in risk stratification and prognosis, as indicated in the present study. These results demonstrate that NGAL and TIMP-1 enhanced the ability of the MEDS score in the risk stratification and prognosis evaluation.

AKI in ICUs is frequently complicated with sepsis, and AKI and sepsis increase mortality synergistically. NGAL is an emerging biomarker for AKI. Its performance in early detection of renal damage has been valuable in several AKI cohorts [21,32,33]. In the present study, 89 (24.7%) septic patients developed AKI, according to the AKIN criteria. NGAL was significantly higher in septic AKI patients than in the other AKI patients and non-AKI patients (*P* <0.01). From the AUC of NGAL for septic AKI, we discovered that the prognostic ability of NGAL was superior to other biomarkers. The present study proved that NGAL was a biomarker for AKI, particularly in septic patients.

### Limitations

Some limitations merit consideration in this study. It was a single-center study and did not compare other severity score systems and biomarkers. Sepsis was established according to criteria for sepsis as defined by ACCP/SCCM as it was difficult to obtain pathogen samples. The dynamic changes of biomarkers were not observed. We did not have information on patient treatment preferences that could introduce confounding and bias.

## Conclusions

NGAL, MMP-9 and TIMP-1 are independent predictors of 28-day mortality in septic patients in the ED, and are promising biomarkers in early diagnosis, risk stratification and evaluation of prognosis in septic patients. NGAL may be a promising biomarker not only in sepsis but also in diagnosis of sepsis with AKI.

## Key messages

- NGAL and TIMP-1 were good biomarkers for reflecting the severity of sepsis and evaluating prognosis of septic patients
- NGAL and TIMP-1 were superior to PCT for predicting 28-day mortality in septic patients in the ED
- NGAL, TIMP-1, MMP-9 and MEDS score were all independent predictors of 28-day mortality in septic patients
- NGAL or TIMP-1 in combination MEDS score enhanced the predictive accuracy of predicting 28-day mortality in septic patients
- NGAL may be a promising biomarker in diagnosis of sepsis with AKI

## Abbreviations

ACCP: American College of Chest Physicians
AKI: acute kidney injury
AKIN: Acute Kidney Injury Network
AUC: area under the ROC curve
ED: emergency department
MEDS: mortality in emergency department sepsis
MMP-9: matrix metalloproteinase-9
NGAL: neutrophil gelatinase-associated lipocalin
NPV: negative predictive value
PCT: procalcitonin
PPV: positive predictive value
ROC: receiver operating characteristic
SCCM: Society of Critical Care Medicine
SIRS: systemic inflammatory response syndrome
TIMP-1: tissue inhibitor of matrix metalloproteinase-1
